# A randomized controlled trial of dapagliflozin on left ventricular hypertrophy in people with type two diabetes: the DAPA-LVH trial

**DOI:** 10.1093/eurheartj/ehaa419

**Published:** 2020-06-24

**Authors:** Alexander J M Brown, Stephen Gandy, Rory McCrimmon, John Graeme Houston, Allan D Struthers, Chim C Lang

**Affiliations:** 1 Division of Molecular & Clinical Medicine, School of Medicine, Ninewells Hospital & Medical School, University of Dundee, Dundee DD1 9SY, UK; 2 Department of Medical Physics, Ninewells Hospital & Medical School, Dundee DD1 9SY, UK; 3 Department of Radiology, Ninewells Hospital & Medical School, Dundee DD1 9SY, UK

**Keywords:** Dapagliflozin, Heart failure, Left ventricular mass, Type 2 diabetes, Insulin resistance

## Abstract

**Aim:**

We tested the hypothesis that dapagliflozin may regress left ventricular hypertrophy (LVH) in people with type 2 diabetes (T2D).

**Methods and results:**

We randomly assigned 66 people (mean age 67 ± 7 years, 38 males) with T2D, LVH, and controlled blood pressure (BP) to receive dapagliflozin 10 mg once daily or placebo for 12 months. Primary endpoint was change in absolute left ventricular mass (LVM), assessed by cardiac magnetic resonance imaging. In the intention-to-treat analysis, dapagliflozin significantly reduced LVM compared with placebo with an absolute mean change of −2.82g [95% confidence interval (CI): −5.13 to −0.51, *P* = 0.018]. Additional sensitivity analysis adjusting for baseline LVM, baseline BP, weight, and systolic BP change showed the LVM change to remain statistically significant (mean change −2.92g; 95% CI: −5.45 to −0.38, *P* = 0.025). Dapagliflozin significantly reduced pre-specified secondary endpoints including ambulatory 24-h systolic BP (*P* = 0.012), nocturnal systolic BP (*P* = 0.017), body weight (*P* < 0.001), visceral adipose tissue (VAT) (*P* < 0.001), subcutaneous adipose tissue (SCAT) (*P* = 0.001), insulin resistance, Homeostatic Model Assessment of Insulin Resistance (*P* = 0.017), and high-sensitivity C-reactive protein (hsCRP) (*P* = 0.049).

**Conclusion:**

Dapagliflozin treatment significantly reduced LVM in people with T2D and LVH. This reduction in LVM was accompanied by reductions in systolic BP, body weight, visceral and SCAT, insulin resistance, and hsCRP. The regression of LVM suggests dapagliflozin can initiate reverse remodelling and changes in left ventricular structure that may partly contribute to the cardio-protective effects of dapagliflozin.

**ClinicalTrials.gov Identifier:**

NCT02956811

**Figure:**
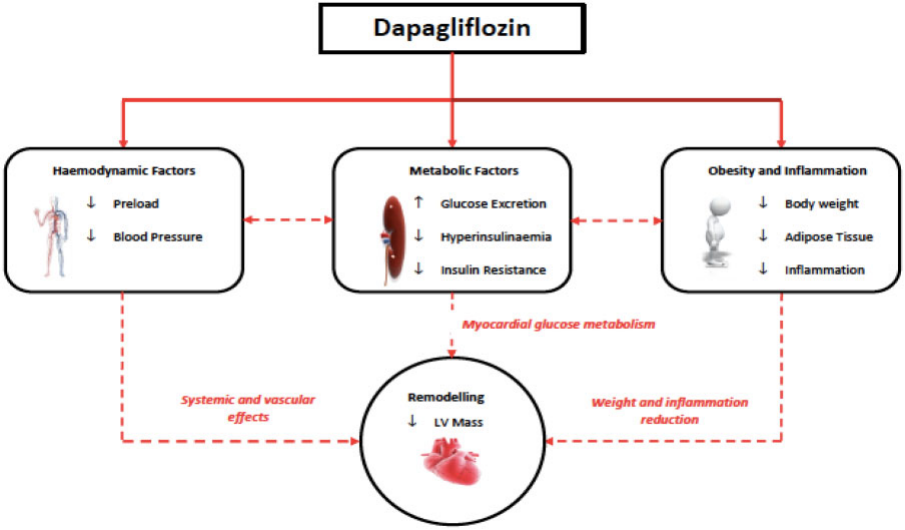

**See page 3433 for the editorial comment on this article (doi: 10.1093/eurheartj/ehaa530)**

## Introduction

Patients with type 2 diabetes (T2D) mellitus have double the risk of cardiovascular death (CVD) compared with people without T2D.^1^^,^^2^ Heart failure is an important manifestation of diabetic heart disease. Men with diabetes are twice as likely to have heart failure as those without T2D and women with T2D have a five-fold increased risk.^3^

Intensive management of hyperglycaemia in people with T2D using oral agents with or without insulin control reduces the risk of microvascular complications but appears to be insufficient to reduce cardiovascular (CV) events.^4–7^ However, the recent Empagliflozin Cardiovascular Outcome Event Trial in Type 2 Diabetes Mellitus Patients (EMPA-REG OUTCOME) trial was a landmark trial as it demonstrated for the first time that a glucose lowering agent could reduce CV events.^8^ The most striking findings of this landmark trial were the profound early effects of the sodium-glucose cotransporter 2 inhibitor (SGLT2i), empagliflozin on CVD, and hospitalization for heart failure (HHF), which were reduced by 38% and 35%, respectively. All-cause mortality was also reduced by 32%. In the Dapagliflozin Effect on Cardiovascular Events (DECLARE TIMI 58) trial, treatment with dapagliflozin was non-inferior to placebo with respect to major adverse cardiovascular events but did result in a lower rate of the other pre-specified primary efficacy outcome (the composite of CVD or HHF) which reflected a lower rate of HHF.^9^ Significant reductions in HHF have also been reported for canagliflozin, in the Canagliflozin Cardiovascular Assessment Study (CANVAS) programme, trial.^10^ These consistent effects of SGLT2i glucose lowering therapy on HHF suggest the benefits may be a class effect and maybe independent of glycaemic control. This is likely to be the case since the Dapagliflozin in Patients With Heart Failure and Reduced Ejection Fraction (DAPA-HF) trial recently reported that dapagliflozin significantly reduced both the incidence of CVD and worsening heart failure in patients with heart failure with reduced ejection fraction, with and without T2D.^11^

The precise mechanisms by which SGLT2i reduces HHF are unclear but may involve natriuresis, reduction in interstitial oedema, reduced preload and afterload, improved renal function and cardio-renal physiology, inhibition of cardiac sodium-hydrogen exchange, and improved cardiac bioenergetics.^12^ The potential reduction on preload and afterload could reduce left ventricular wall stress and facilitate beneficial cardiac remodelling. Cardiac remodelling can be achieved through regression of left ventricular hypertrophy (LVH). Left ventricular hypertrophy is highly prevalent amongst people with T2D with a reported prevalence of up to 70%, and the pathophysiology of LVH in T2D is not fully understood as it can develop independently of blood pressure (BP).^13^^,^^14^ The pathophysiology of LVH in T2D is complex. In addition to risk factors seen in people without T2D both obesity and associated insulin resistance are also associated with LVH in T2D.^15–20^ Importantly, LVH is a strong independent predictor of CVD and CV events.^21^^,^^22^

In this ‘proof of concept’ randomized controlled trial, we hypothesized that dapagliflozin would cause regression of left ventricular mass (LVM) in people with T2D and LVH assessed using cardiac magnetic resonance (CMR) imaging. If dapagliflozin can cause regression of LVM, we wish to try to better understand the likely mechanisms. Therefore, we also studied, as exploratory secondary outcomes, the drug’s effect on body weight and composition, BP and insulin resistance that are all potentially implicated in the pathophysiology of LVH in T2D (Supplementary material online, *Figure S1*).

## Methods

The original design and methods of the DAPA-LVH trial has been published previously.^23^

### Study design

The DAPA-LVH study (NCT02956811) was a single-centre, double-blind, placebo-controlled trial designed to evaluate the efficacy of dapagliflozin 10 mg once daily treatment compared with placebo on LVH in participants with T2D identified to have LVH. The study was approved by the East of Scotland Research Ethics Committee (16/ES/0131) and all participants provided written informed consent to participate in the study and were enrolled in this trial for a period of 10–12 months. Supplementary material online, *Figures S2* and *S3* show the DAPA-LVH trial study design flow chart and consort diagram. Supplementary material online, *Table S1* shows all the assessments made at each trial visit.

### Study participants

The study population included 66 participants recruited between February 2017 and May 2018 from Tayside, Scotland using research databases, hospital records, and local general practices.

Participants were aged 18–80 years and had been previously diagnosed with T2D based on the American Diabetes Association guidelines. Presence of LVH was defined using echocardiography as either LV mass index of >115 g/m^2^ for men and >95 g/m^2^ for women indexed to body surface area (BSA) or >48 g/m^2.7^ or 44 g/m^2.7^ when indexed to height.^2.7^ People with hypertension were not excluded from the study but their clinic BP had to be <145/90 mmHg (mean value of three measurements performed at 5-min intervals on the same arm). If any individual had borderline office measurements an ambulatory BP monitor was performed to ensure BP adequately controlled. Participants had to have an HbA1c measurement within the last 6 months at screening between 48 and 85 mmol/mol. In this ‘proof of concept’ study, the primary endpoint of interest is LVM as assessed by magnetic resonance imaging (MRI). We have focused to explore this in a defined population of patients with LVH with no clinical heart failure.

Participants who met the eligibility criteria were randomly assigned to receive either dapagliflozin 10 mg once daily or matching placebo in a double-blind fashion.

### Magnetic resonance imaging

Baseline and final (after a minimum of 10–12 months) MRI scans were performed on a 3T PrismaFIT MRI scanner (Siemens, Erlangen, Germany) using body array and spine matrix radiofrequency coils. Both the cardiac and abdominal MRI protocols are described in detail in Section A in the Supplementary material online. Both the cardiac and abdominal MRIs were analysed by a single-blinded observer.

### Echocardiogram

The echocardiograms were done using a Phillips Epiq 7 machine. Screening for LVH was performed as per the American Society of Echocardiography (ASE) guidelines.^24^ All the echocardiograms were performed by a single-blinded observer with British Society of Echocardiography accreditation in transthoracic echocardiography.

### Laboratory investigations

Routine biochemical and haematological investigations were measured at all study visits as well as safety parameters. Biomarkers of ventricular wall stress (Amino-terminal pro B-type natriuretic peptide (NT-proBNP; Multi array, Meso Scale Discovery, Mesoscale Diagnostics, USA), oxidative stress (myeloperoxidase; R&D Systems Quantikine Human MPO Immunoassay), inflammation (high-sensitivity C-reactive protein; Kalon High-Sensitivity CRP assay), fasting Insulin (ALPCO Insulin ELISA), leptin (the R&D Systems Quantikine Human Leptin Immunoassay), and N-terminal Procollagen III peptide (Cloud Clone Procollagen III N-Terminal Propeptide competitive inhibition enzyme immunoassay) were measured at baseline and at the final visit. Homeostatic Model Assessment of Insulin Resistance (HOMA-IR) was calculated according to the formula: [(fasting insulin (uIU/mL) × fasting glucose (mmol/L) × 18)]/22.5. Vital signs (office BP, heart rate, weight, hip, and waist circumference) were assessed at every study visit. Safety of dapagliflozin was also assessed in this patient population. All outcome parameters were measured at randomization and final visits, except safety parameters which were measured in all in-person visits.

### Study endpoints

The primary endpoint was to determine whether dapagliflozin induces regression in absolute LVM assessed by cardiac MRI. The secondary endpoints were changes in LVM index (LVMi) indexed to BSA, height^1.7^and height.^2.7^ Other exploratory secondary endpoints included changes in LV ejection fraction, LV volumes; abdominal obesity assessed by MRI; BP assessed by 24-hambulatory measurement, weight, glycaemic parameters and blood biomarkers.

### Power calculation

The power calculation of the primary outcome, absolute change in LV mass determined by cardiac MRI, was based on two previous studies.^25^^,^^26^ One study examined LVM regression in participants with ischaemic heart disease and reported that allopurinol significantly reduced LVM by −5.2 ± 5.8 g compared with placebo [−1.3 ± 4.5 g (*P* < 0·007)].^25^ This degree of LVH regression was similar to that reported in the echo sub-study of the LIFE study.^27^ For an 80% power at a 5% significance level (*α* = 0·05), to detect a similar change in absolute LVM of 5 g, we required 29 subjects per group. To allow for a potential 10% dropout rate, the study aimed to recruit a minimum of total of 64 participants (32 per group). The 10% dropout rate is standard for such studies and includes those who withdraw consent.

### Statistical analysis

The primary outcome comparison was based on intention-to-treat (ITT) analysis, i.e. all participants who had baseline measurements and took at least one dose of investigational medicinal product were analysed as part of the group to which they were randomized. Missing post-baseline values was imputed using the baseline observation carried forward method. In addition to this to provide a true estimate of the efficacy of intervention, a per-protocol analysis was also performed. The comparison between intervention and placebo groups was compared using independent samples *t*-tests for continuous variables and χ^2^ test for dichotomous variables. Continuous variables with normal distribution are presented as mean (SD). Non-normally distributed data are presented as medians alongside their interquartile ranges (IQR). Additionally, we performed a sensitivity analysis using analysis of covariance (ANCOVA) model to evaluate the robustness of treatment with change in LVM and treatment as fixed effects, and baseline values for LVM, body weight, systolic blood pressure (SBP), diastolic blood pressure (DBP), and SBP change as covariates. Sensitivity analysis was also performed for the ambulatory BP measurements with the SBP change as the dependent variable and the baseline BP was the covariate and an ANCOVA was carried out. A *P*-value <0.05 was considered significant. Data were analysed using SPSS 22.0 (IBM Corp, Armonk, NY, USA).

## Results

Of the 320 participants who were screened, 66 subjects fulfilled all the study criteria and were randomly allocated to receive either dapagliflozin (*n* = 32) or placebo (*n* = 34). Supplementary material online, *Figure S3* shows the DAPA-LVH trial consort diagram.

Sixty-two participants completed the study (*n* = 29 in dapagliflozin group; *n* = 33 in placebo group). Four people withdrew from the study early; breast cancer (*n* = 1), unable to obtain holiday insurance as participating in a clinical trial (*n* = 1), hyponatraemia (*n* = 1) and claustrophobia thus unable to complete the final MRI. These people however were included in our ITT analysis.

### Patient characteristics

The baseline characteristics of the participants at randomization are shown in *Tables 1 and**2*. When comparing the two groups, apart from serum potassium there were no significant differences at baseline. 

**Table 1:** Baseline characteristics

Variable	Total cohort	Dapagliflozin	Placebo	*P*-value
Participants randomized	66	32	34	
Demographics				
Age (years)	65.53 ± 6.87	64.25 ± 7.01	66.74 ± 6.62	0.143
Male	38 (57.6%)	20 (62.5%)	18 (52.9%)	0.432
Never smoked	31 (47.0%)	14 (43.8%)	17 (50.0%)	0.611
Current smoker	4 (6.1%)	3 (9.4%)	1 (2.9%)	0.348
Ex—smoker	31 (47.0%)	15 (46.9%)	16 (47.1%)	0.988
Duration of diabetes (years)^a^	10.0 (6.0, 15.0)	8.5 (5.25, 14.5)	10.0 (7.5, 15.0)	0.343
Weight (kg)	91.53 ± 14.26	91.58 ± 14.62	91.48 ± 14.13	0.977
BMI (kg/m^2^)	32.45 ± 4.41	32.30 ± 4.66	32.59 ± 4.22	0.793
Co-morbidities				
IHD	8 (12.1%)	2 (6.3%)	6 (17.6%)	0.260
Hypertension	51 (77.3%)	26 (81.3%)	25 (73.5%)	0.454
Stroke	7 (10.6%)	1 (3.1%)	6 (17.6%)	0.106
Hypercholesterolaemia	38 (57.6%)	17 (53.1%)	21 (61.8%)	0.478
Medications				
Ace inhibitor	35 (53.0%)	17 (53.1%)	18 (52.9%)	0.988
Angiotensin receptor blocker	11 (16.7%)	5 (15.6%)	6 (17.6%)	0.826
Calcium channel blocker	22 (33.3%)	9 (28.1%)	13 (38.2%)	0.384
Thiazide diuretic	13 (19.7%)	9 (28.1%)	4 (11.8%)	0.095
Beta-blocker	9 (13.6%)	4 (12.5%)	5 (14.7%)	0.794
Alpha-blocker	7 (10.6%)	4 (12.5%)	3 (8.8%)	0.705
Aspirin	10 (15.2%)	4 (12.5%)	6 (17.6%)	0.734
Clopidogrel	7 (10.6%)	2 (6.3%)	5 (14.7%)	0.428
Statin	55 (83.3%)	25 (78.1%)	30 (88.2%)	0.271
Metformin	66 (100.0%)	32 (100.0%)	34 (100.0%)	Constant
Sulphonlylurea	15 (22.7%)	7 (21.9%)	8 (23.5%)	0.873
DDP-IV inhibitor	7 (10.6%)	4 (12.5%)	3 (8.8%)	0.705
GLP-1 agonist	7 (10.6%)	4 (12.5%)	3 (8.8%)	0.705
Thiazolidinedione	3 (4.5%)	0 (0.0%)	3 (8.8%)	0.239
Insulin	14 (21.2%)	7 (21.9%)	7 (20.6%)	0.898
Blood pressure				
24 h SBP baseline^b^	129.02 ± 10.09	130.41 ± 9.62	127.67 ± 10.65	0.281
	(*n* = 65)		(*n* = 33)	
24 h DBP baseline^b^	73.42 ± 7.04	74.41 ± 7.88	72.46 ± 6.09	0.267
	(*n* = 65)		(*n* = 33)	
Heart rate baseline^c^	75.31 ± 13.91	74.44 ± 13.9	76.15 ± 14.08	0.623
	(*n* = 65)		(*n* = 33)	
Daytime SBP baseline^b^	131.43 ± 10.74	132.59 ± 10.37	130.30 ± 11.19	0.394
	(*n* = 65)		(*n* = 33)	
Daytime DBP baseline^b^	75.37 ± 7.37	76.44 ± 8.57	74.33 ± 5.94	0.253
	(*n* = 65)		(*n* = 33)	
Nocturnal SBP baseline^d^	120.50 ± 12.06	123.84 ± 11.1	119.81 ± 12.8	0.183
	(*n* = 64)		(*n* = 32)	
Nocturnal DBP baseline^d^	67.50 ± 7.77	68.97 ± 7.84	66.00 ± 7.52	0.127
	(*n* = 64)		(*n* = 32)	
Office SBP baseline	136.68 ± 8.32	137.25 ± 7.5	136.15 ± 9.11	0.594
Office DBP baseline	78.45 ± 8.4	79.16 ± 8.63	77.79 ± 8.25	0.514
Laboratory measurements				
Haemoglobin (g/L)	138.36 ± 12.72	138.31 ± 13.61	138.41 ± 12.03	0.514
Haematocrit (%)	41.73 ± 3.31	41.46 ± 3.30	41.99 ± 3.35	0.975
Creatinine (umol/L)	68.11 ± 18.38	65.09 ± 16.36	70.94 ± 19.92	0.199
GFR (mL/min/1.73^2^)	101.88 ± 27.06	107.53 ± 25.40	96.56 ± 27.86	0.100
Sodium (mmol/L)	138.92 ± 2.24	138.72 ± 2.16	139.12 ± 2.33	0.474
Potassium (mmol/L)	4.34 ± 0.35	4.23 ± 0.32	4.44 ± 0.35	0.013
Fasting glucose (mmol/L)	8.05 ± 2.96	7.80 ± 3.50	8.05 ± 3.00	0.964
Fasting insulin (uIU/mL)^a^^,e^	11.08	10.56	11.38 ± 11.42	0.521
(*n* = 48)	(7.43, 18,93)	(6.30, 18.99)	(7.90, 19.320	
		(*n* = 22)	(*n* = 26)	
HOMA-IR^a^^,e^	4.03	4.03 ± 4.26	3.91	0.756
(*n* = 48)	(2.75, 6.78)	(2.41, 6.67)	(2.96, 7.37)	
		(*n* = 22)	(*n* = 26)	
HbA1c (mmol/mol)	60.94 ± 10.61	61.75 ± 11.19	60.18 ± 10.15	0.551
NT-proBNP (pg/mL)^a^	274.42	217.98	365.03	0.218
	(116.12, 568.45)	(82.93, 560.56)	(144.86, 678.12)	
Leptin (pg/mL)^a^	15.65	13.12	17.92	0.124
	(7.48, 30.75)	(5.69, 29.10)	(10.71, 38.94)	
Myeloperoxidase (ng/mL)^a^	117.66	129.14	114.37	0.837
	(64.83, 246.42)	(59.74, 278.11)	(65.03, 216.40)	
NT pro collagen III (ng/mL)^a^	16.60	15.91	17.25	0.878
	(13.42, 20.74)	(13.69, 21.59)	(13.10, 20.74)	
hsCRP (ng/mL)^a^	1696.30	1168.55	2225.01	0.349
	(687.10, 3966.83)	(635.62, 4685.52)	(795.84, 3966.83)	

Data are mean ± SD, *n* (%).

BSA, body surface area; DBP, diastolic blood pressure; DDP-IV; dipeptidyl peptidase-4; GFR, glomerular filtration rate; GLP-1, glucagon like peptide; HDL, high-density lipoprotein; HOMA-IR, homeostatic model assessment of insulin resistance; hsCRP, high sensitive C-reactive protein; IHD, ischaemic heart disease; LDL, low-density lipoprotein; NT-proBNP, N-terminal pro natriuretic peptide; SBP, systolic blood pressure.

a Median (quartile 1, quartile 3).

b One patient unable to tolerate ABPM.

c Heart rate taken from ambulatory 24-h recording.

d Further patient unable to tolerate nocturnal ABPM.

e Only performed on people not on insulin.

**Table 2:** Baseline MRI measurements

Variable	Total cohort	Dapagliflozin	Placebo	*P*-value
Participants randomized	66	32	34	
Absolute LV mass (g)	123.96 ± 22.46	126.47 ± 20.54	121.61 ± 24.20	0.383
LV mass index BSA (g/m^2^)	59.95 ± 8.26	60.92 ± 7.76	59.04 ± 8.73	0.360
EF (%)	71.94 ± 5.86	71.31 ± 5.42	72.54 ± 6.27	0.398
EDV (mLs)	124.04 ± 24.07	127.63 ± 22.54	120.66 ± 25.29	0.243
ESV (mLs)	35.34 ± 10.63	37.17 ± 9.92	33.63 ± 11.13	0.178
SV (mLs)	88.42 ± 17.65	90.45 ± 16.36	87.03 ± 18.88	0.435
Left atrial area	23.91 ± 5.25	24.73 ± 5.86	23.13 ± 4.55	0.218
VAT volume (cm^3^)^a^	6372.55 ± 2038.19	6301.79 ± 1988.24	6437.06 ± 2110.43	0.792
	(*n* = 65)	(*n* = 31)		
SCAT volume (cm^3^)^a^	9135.8 ± 3425.26	9058.34 ± 3857.04	9213.27 ± 2994.46	0.860
	(*n* = 62)	(*n* = 31)	(*n* = 31)	
VAT/SCAT volume ratio^a^	0.77 ± 0.33 (*n* = 62)	0.79 ± 0.31 (*n* = 31)	0.74 ± 0.35 (*n* = 62)	0.583

Data are mean ± SD, *n* (%).

EDV, end-diastolic volume; EF, ejection fraction; ESV, end-systolic volume; LV, left ventricular; LVM, left ventricular mass; LVMI, left ventricular mass index; MRI, magnetic resonance imaging; SCAT, subcutaneous adipose tissue; SV, stroke volume; VAT, visceral adipose tissue.

a Some scans removed due to artefact making accurate VAT or SCAT measurement not possible – see text for details.

### Primary outcome

#### Effect of dapagliflozin on LVM

**Figure 1:**
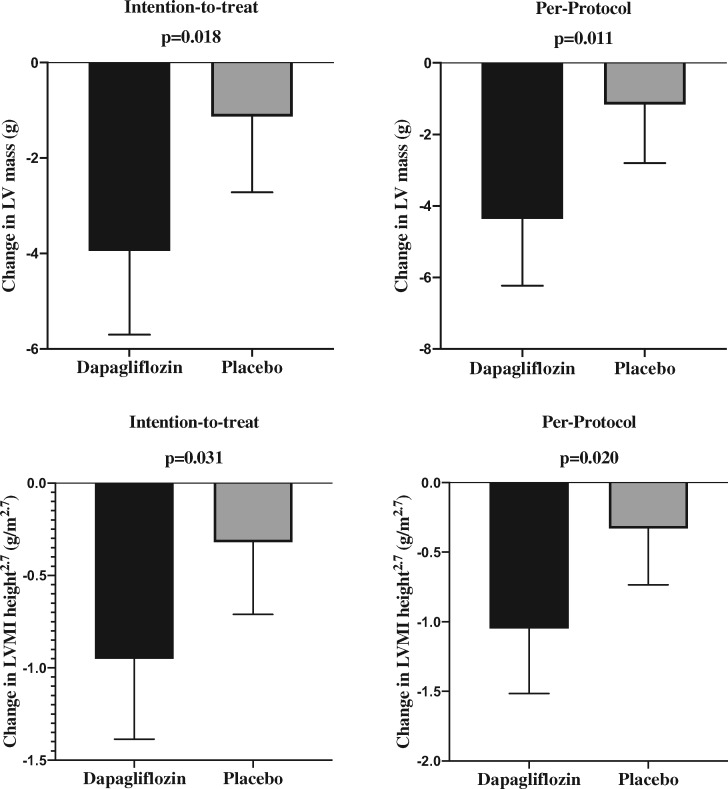
Column bar charts showing the mean regression of left ventricular mass and left ventricular mass index height^2.7^ following dapagliflozin treatment.

After a mean treatment period of almost 12 months dapagliflozin reduced LVM as measured by MRI in the ITT analysis (change in LVM: dapagliflozin group −3.95 ± 4.85 g vs. placebo group −1.13 ± 4.55 g; *P* = 0.018), leading to an absolute mean difference of −2.82 g [95% confidence interval (CI): −5.13 to −0.51]. The reduction in LVM was even greater in the per-protocol population (change in LVM: dapagliflozin group −4.36 ± 4.92 g vs. placebo group −1.17 ± 4.43 g; *P* = 0.011), leading to an absolute mean difference of −3.20 g (95% CI: −5.62 to −0.77) (*Table 3*; *Figure 1*). 

**Take home figure:**
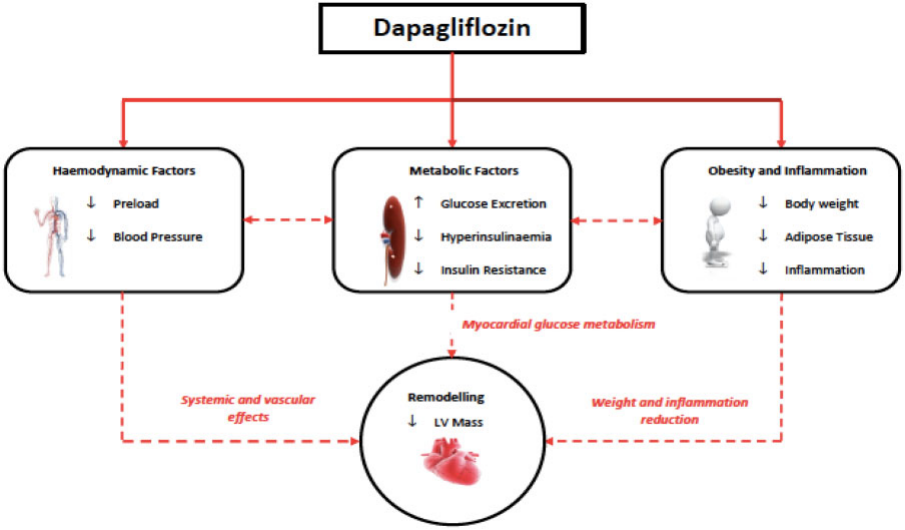
Proposed mechanisms by which dapagliflozin regressed left ventricular mass.

**Table 3:** Changes in parameters measured by cardiac magnetic resonance after 12 months dapagliflozin treatment

Variable	Intention-to-treat analysis				Per-protocol analysis			
	Dapagliflozin	Placebo	Difference^a^	*P*-Value	Dapagliflozin	Placebo	Difference^a^	*P*-Value
	(*n* = 32)	(*n* = 34)	(95% CI)		(*n* = 29)	(*n* = 33)	(95% CI)	
Primary endpoint								
Absolute LVM (g)	−3.95 ± 4.85	−1.13 ± 4.55	−2.82 (−5.13 to −0.51)	**0.018**	−4.36 ± 4.92	−1.17 ± 4.43	−3.2 (−5.62 to −0.77)	**0.011**
Secondary endpoints								
LVMI BSA (g/m^2^)	−0.58 ± 2.29	−0.38 ± 1.79	−0.20 (−1.21 to 0.80)	0.691	−0.64 ± 2.40	−0.39 ± 1.81	−0.25 (−1.32 to 0.82)	0.644
LVMI Height (g/m)	−2.33 ± 2.87	−0.71 ± 2.68	−1.62 (−2.99 to −0.26)	**0.021**	−2.57 ± 2.91	−0.73 ± 2.72	−1.84 (−3.27 to −0.41)	**0.013**
LVMI Height^1.7^ (g/m^1.7^)	−1.61 ± 2.00	−0.51 ± 1.87	−1.09 (−2.05 to −0.15)	**0.024**	−1.78 ± 2.03	−0.52 ± 1.89	−1.25 (−2.25 to −0.25)	**0.015**
LVMI Height^2.7^ (g/m^2.7^)	−0.95 ± 1.20	−0.32 ± 1.12	−0.63 (−1.21 to −0.06)	**0.031**	−1.05 ± 1.22	−0.33 ± 1.14	−0.72 (−1.32 to−0.12)	**0.020**
EF (%)	1.45 ± 4.08	0.66 ± 3.76	0.79 (−1.14 to 2.72)	0.415	1.60 ± 4.26	0.68 ± 3.81	0.92 (−1.13 to 2.97)	0.372
EDV (mLs)	−0.15 ± 11.59	1.44 ± 10.62	−1.59 (−7.06 to 3.87)	0.562	−0.17 ± 12.20	1.48 ± 10.78	−1.65 (−7.49 to 4.18)	0.573
ESV (mLs)	−1.86 ± 4.83	−0.74 ± 4.81	−1.12 (−3.50 to 1.25)	0.348	−2.05 ± 5.04	−0.76 ± 4.89	−1.29 (−3.82 to 1.23)	0.310
SV (mLs)	1.71 ± 11.18	2.18 ± 10.45	−0.47 (−5.79 to 4.85)	0.860	1.88 ± 11.75	2.24 ± 10.60	−0.36 (−6.04 to 5.32)	0.900
Left atrial area (Cm^2^)^b^	−0.25 ± 3.38	0.00 ± 3.5	−1.20 (−2.82 to 0.42)	0.143	−0.5 ± 3.75	0.0 ± 3.5	−1.29 (−3.01 to 0.44)	0.088

*P*-values in bold indicate <0.05.

BSA, body surface area; EDV, end-diastolic volume; EF, ejection fraction; ESV; end-systolic volume; LVM, left ventricular mass; LVMI, left ventricular mass index; SV, stroke volume.

a Absolute mean difference between groups. All values expressed in mean ± SD unless stated.

b Median ± IQR.

Following sensitivity analysis, the reduction in LVM remained greater in the dapagliflozin group compared with placebo: (i) for the ITT arm—estimated marginal means: dapagliflozin group, −4.00 g (95% CI: −5.75 to −2.26) vs. placebo group −1.09 g (95% CI: −2.77 to 0.60) and (ii) per-protocol population—estimated marginal means: dapagliflozin group, −4.43 g (95% CI: −6.29 to −2.58) vs. placebo group −1.11 g (95% CI: −2.84 to 0.62) and remained statistically significant (*P* = 0.025 for ITT and *P* = 0.011 for per-protocol analysis), suggesting that this finding was robust and not driven by potential relevant baseline characteristics (Supplementary material online, *Table S2*).

Dapagliflozin induced greater LVH regression in those with an above median LVMI at baseline, as might be expected (mean change of −3.88 g (95% CI −7.15 to −0.61, *P* = 0.021) (Supplementary material online, *Table S3*).

### Secondary outcomes

#### Cardiovascular measures

##### Effect of dapagliflozin on indexed left ventricular mass

Dapagliflozin resulted in significant reductions in LVM indexed to height, height^1.7 ^and height^2.7^ in both the ITT and per-protocol populations (*Table 3*).

This remained the case following sensitivity analysis correcting for the same confounders discussed above (Supplementary material online, *Table S2*).

With the reduction in body weight dapagliflozin did not reduce LMVI to BSA in either the ITT or per protocol population (Table 3). However, when LVM was indexed to baseline BSA dapagliflozin treatment was significant (change in LVMI BSA: dapagliflozin group −2.06 g/m^2^ vs. placebo group −0.65 g/m^2^; *P* = 0.019) leading to an estimated mean difference of −2.41 g/m^2^ (95% CI: −2.58 to −0.24).

CMR-measured end-diastolic volume, end-systolic volume, left ventricular ejection fraction, stroke volume did not change significantly with dapagliflozin therapy (Table 3).

##### Effect of dapagliflozin on blood pressure

In both ITT and per-protocol analyses, dapagliflozin significantly reduced 24-h ambulatory SBP and nocturnal systolic BP (Table 4) (Supplementary material online, *Figure S4*). In the ITT analysis, dapagliflozin resulted in a mean difference in 24-h ambulatory SBP of −3.6 mmHg (95% CI: −6.4 to −0.8; *P* = 0.012). Dapagliflozin also resulted in a mean difference in nocturnal systolic BP of −4.4 mmHg (95% CI: to – 7.9 to −0.8; *P* = 0.017). These changes remained significant after correction for baseline BP measurements (Supplementary material online, *Table S4*). 

**Table 4:** Changes in blood pressure after 12 months of dapagliflozin treatment

Variable change	Intention-to-treat analysis				Per-protocol analysis			
	Dapagliflozin	Placebo	Difference^a^	*P*-value	Dapagliflozin	Placebo	Difference^a^	*P*-value
	(*n* = 32)	(*n* = 34)	(95% CI)		(*n* = 29)	(*n* = 33)	(95% CI)	
24 h SBP^b^	−2.78 ± 5.94	0.85 ± 5.40 (*n* = 33)	−3.63 (−6.44 to −0.82)	**0.012**	−3.07 ± 6.18	0.88 ± 5.48 (*n* = 32)	−3.94 (−6.93 to −0.96)	**0.011**
24 h DBP^b^	−0.94 ± 3.98	0.06 ± 4.87 (*n* = 33)	−0.1 (−3.2 to 1.21)	0.370	−1.03 ± 4.18	0.06 ± 4.94 (*n* = 32)	−1.1 (−3.46 to 1.260	0.356
Heart rate^b^–d	−2.00 ± 5.75	1.00 ± 8.50 (*n* = 33)	−2.1 (−5.64 to 1.43)	0.184	−2.0 ± 7.5	1.0 ± 8.80 (*n* = 32)	−2.27 (−6.05 to 1.51)	0.183
Daytime SBP^b^	−2.47 ± 6.56	0.55 ± 6.45 (*n* = 33)	−3.01 (−6.24 to 0.21)	0.066	−2.72 ± 6.85	0.56 ± 6.55 (*n* = 32)	−3.29 (−6.72 to 0.15)	0.060
Daytime DBP^b^	−1.03 ± 5.18	0.24 ± 5.80 (*n* = 33)	−1.27 (−4.00 to 1.46)	0.355	−1.14 ± 5.44	0.25 ± 5.90 (*n* = 32)	−1.39 (−4.30 to 1.53)	0.345
Nocturnal SBP^e^	−3.47 ± 7.54	0.91 ± 6.70 (*n* = 32)	−4.38 (−7.94 to −0.81)	**0.017**	−3.83 ± 7.84	0.94 ± 6.81 (*n* = 31)	−4.76 (−8.55 to −0.98)	**0.015**
Nocturnal DBP^e^	−2.25 ± 5.90	0.16 ± 4.14 (*n* = 32)	−2.41 (−4.95 to 0.14)	0.063	−2.48 ± 6.16	0.16 ± 4.20 (*n* = 31)	−2.64 (−5.35 to 0.06)	0.059
Office SBP	−5.28 ± 8.63	−1.79 ± 7.26	−3.49 (−7.40 to 0.43)	0.080	−5.83 ± 8.89	−1.85 ± 7.37	−3.98 (−8.11 to 0.15)	0.059
Office DBP	−2.97 ± 5.62	−2.24 ± 7.48	−0.73 (−4.00 to 2.54)	0.656	−3.27 ± 5.82	−2.30 ± 7.58	−0.97 (−4.39 to 2.44)	0.577

*P*-values in bold indicate <0.05.

DBP, diastolic blood pressure; SBP, systolic blood pressure.

a Absolute mean Difference between groups. All other values expressed in mean ± SD unless stated.

b One participant unable to tolerate any ambulatory blood pressure monitoring.

c Median ± IQR.

d Twenty-four hour heart rate recorded during ambulatory blood pressure monitoring.

eOne further participant unable to tolerate overnight blood pressure monitoring.

There was an observed moderate correlation between change in LVM and change in ambulatory 24 SBP and nocturnal SBP with *r* = 0.415, *n* = 61, *P* = 0.001, and *r* = 0.321, *n* = 60, *P* = 0.012, respectively.

There were only four changes in total to the antihypertensive with two dose reductions in the dapagliflozin arm and one dose reduction and one dose increase in the placebo arm.

### Metabolic outcomes

#### Effect of dapagliflozin on obesity parameters

The ITT analysis consisted of 65 participants where complete visceral adipose tissue (VAT) volumes were available for analysis (31 and 34 in dapagliflozin arm and placebo arm, respectively) and 62 where complete subcutaneous adipose tissue (SCAT) volumes were available for analysis (31 in each arm). One participant was unable to complete the abdominal MRI at the final visit due to claustrophobia. Therefore, in the per-protocol population there were 60 participants where complete VAT volumes were available for analysis (28 and 32 in dapagliflozin and placebo arm, respectively, and 57 participants where complete SCAT volumes were available for analysis (28 and 29 in dapagliflozin and placebo arm, respectively).

In both the ITT and the per-protocol population dapagliflozin treatment significantly reduced VAT and SCAT (Table 5) (Supplementary material online, *Figure S5*). 

**Table 5:** Changes in obesity parameters after 12 months dapagliflozin treatment

Variable change	Intention-to-treat analysis				Per-protocol analysis			
	Dapaglilflozin	Placebo	Difference^a^	*P*-value	Dapaglilflozin	Placebo	Difference^a^	*P*-value
	(*n* = 32)	(*n* = 34)	(95% CI)		(*n* = 29)	(*n* = 33)	(95% CI)	
Weight (kg)	−4.27 ± 2.50	−0.50 ± 2.19	−3.77 (−4.92 to −2.61)	**<0.001**	−4.56 ± 2.41	−0.52 ± 2.22	−4.03 (−5.21 to −2.86)	**<0.001**
BMI (kg/m^2^)^b^	−1.53 ± 0.93	−0.17 ± 0.74	−1.35 (−1.77 to −0.94)	**<0.001**	−1.63 ± 0.91	−0.18 ± 0.75	−1.45 (−1.87 to −1.03)	**<0.001**
VAT volume (cm^3^)^c^	−565.17 ± 691.27	114.22 ± 593.69	−679.4	**<0.001**	−625.73 ± 701.18	121.36 ± 611.81	−747.09	**<0.001**
	(*n* = 31)		(−998.00 to −360.80)		(*n* = 28)	(*n* = 32)	(−1086.34 to −407.84)	
SCAT volume (cm^3^)^c^	−720.84 ± 687.83	−111.08 ± 643.42	−609.76	**0.001**	−798.07 ± 679.52	−118.74 ± 665.30	−679.33	**<0.001**
	(*n* = 31)	(*n* = 31)	(−948.13 to −271.28)		(*n* = 28)	(*n* = 29)	(−1036.47 to −322.19)	
VAT/SCAT volume ratio^c^	(*n* = 31)	0.02 ± 0.06	−0.03 (−0.06 to 0.00)	**0.023**	−0.01 ± 0.06	0.021 ± 0.057	−0.04 (−0.07 to −0.01)	**0.023**
	−0.01 ± 0.06	(*n* = 31)			(*n* = 28)	(*n* = 29)		

*P*-values in bold indicate <0.05.

BMI, body mass index; SCAT, subcutaneous adipose tissue; VAT, visceral adipose tissue.

a Absolute mean difference between groups. All values expressed in mean ± SD unless stated.

b Median ±IQR.

c Some scans removed due to artefact making accurate VAT or SCAT measurement not possible—see text for details.

This also meant dapagliflozin significantly reduced the VAT/SCAT ratio in both the ITT (*P* = 0.023) and the per-protocol (*P* = 0.023) populations. There was an observed strong correlation between change in LVM and change in VAT, *r* = 0.592, *n* = 60, *P* < 0.001, and moderate correlation between change in LVM and change in SCAT *r* = 0.360, *n* = 57, *P* = 0.006.

Compared with placebo in both analyses dapagliflozin treatment resulted in significant reduction in weight. Mixed model analysis of the per-protocol population showed the weight loss effect to be most significant with the first 4–6 months of treatment (Supplementary material online, *Figure S6*).

#### Effect of dapagliflozin on blood parameters

In this study, 11.9 months dapagliflozin therapy increased both haemoglobin and haematocrit from baseline. Dapagliflozin reduced fasting glucose, glycated haemoglobin and improved HOMA-IR, and reduced hsCRP compared with placebo (Table 6). 

**Table 6:** Changes in safety and research blood parameters after 12 months dapagliflozin treatment

Variable	Intention-to-treat analysis			
	Dapagliflozin	Placebo	Difference^a^	*P*-value
	(*n* = 32)	(*n* = 34)	(95%CI)	
Haemoglobin (g/L)	7.00 ± 11.75	−2.00 ± 5.00	9.51 (5.85 to 13.18)	**<0.001**
Haematocrit (%)	2.60 ± 0.02	0.30 ± 0.02	2.90 (1.84 to 3.96)	**<0.001**
Creatinine (umol/L)	1.34 ± 5.89	−0.91 ± 5.83	2.26 (−0.63 to 5.14)	0.123
cGFR (mL/min/1.732)	−1.16 ± 10.48	1.59 ± 7.19	−2.74 (−7.14 to 1.65)	0.217
Sodium (mmol/lL)	−0.75 ± 2.05	0.38 ± 1.83	−1.13 (−2.09 to 0.18)	0.121
Potassium (mmol/L)	−0.03 ± 0.26	−0.04 ± 0.30	−0.01 (−0.12 to 0.15)	0.852
Fasting glucose (mmol/L)	−1.06 ± 2.08	0.62 ± 2.11	−1.68 (−2.71 to −0.65)	**0.002**
HbA1c (mmol/mol)	−6.28 ± 8.25	−0.79 ± 10.89	−5.49 (−10.26 to −0.71)	**0.025**
NT-proBNP (pg/mL)^b^	7.14 ± 138.69	40.19 ± 219.47	−103.68 (−326.90 to 119.54)	0.551
Leptin (pg/mL)^b^	−447.55 ± 5299.58	477.6 ± 6314.88	−2931.7 (−6901.46 to 1038.07)	0.256
Myeloperoxidase (ng/mL)^b^	0.00 ± 107.04	−36.49 ± 85.63	23.02 (−31.05 to 77.08)	0.172
NT-pro collagen III (ng/mL)	−0.44 ± 5.06	−0.1 ± 4.24	−0.46 (−2.20 to 1.29)	0.653
hsCRP (ng/L)^b^	−163.73 ± 1040.76	66.73 ± 1258.37	−1296.04 (−2650.59 to −31.50)	**0.049**
Fasting insulin (uU/mL) (*n* = 48)^b^,c	−2.34 ± 5.59	−0.58 ± 7.14	−3.61 (−6.97 to −0.26)	0.098
	(*n* = 22)	(*n* = 26)		
HOMA-IR (*n* = 48)^b^,c	−2.1 ± 2.37 (*n* = 22)	0.46 ± 3.23 (*n* = 26)	−2.56 (−4.47 to −0.65)	**0.017**

*P*-values in bold indicate <0.05.

eGFR, estimated glomerular filtration rate; HDL, high-density lipoprotein; HOMA-IR, homeostatic model assessment of insulin resistance; hsCRP, high sensitive C-reactive protein; LDL, low-density lipoprotein; NT-proBNP, N-terminal pro natriuretic B-type natriuretic peptide.

a Absolute mean difference between groups. All other values expressed in mean ± SD unless stated.

b Median ± IQR.

c Only performed on the participants not on insulin.

### Tolerability and safety of dapagliflozin

In total, there were 169 adverse events, 86 events in the dapagliflozin arm and 83 in the placebo arm although most of these were transient and mild to moderate in severity. There were no reported cases of diabetic ketoacidosis. There were five serious adverse events recorded during the trial (two in the dapagliflozin arm and three in the placebo arm). The incidence of common side effects reported with SGLT2i is illustrated in Supplementary material online, *Table S5*.

## Discussion

The main finding of our study is that following 1-year of dapagliflozin (10 mg) there were significant reductions in CMR-measured LVM in normotensive T2D participants who had LVH at baseline. Dapagliflozin was also shown to significantly reduce measures of body weight and VAT, 24-h ambulatory and nocturnal SBP and insulin resistance that maybe implicated in the pathophysiology of LVH in T2D.

To the best of our knowledge, this is the first randomized controlled trial investigating the effect of dapagliflozin on LVH in patients with T2D. We found that dapagliflozin reduced LVM by 3.95 g when compared to a reduction of 1.13 g in the placebo group. The small reduction in LVM observed in the placebo group in our study is not unexpected and is often reported in clinical trials. This is likely because our clinical participants were closely monitored at all trial visits to ensure adequate BP and glycaemic control. This close monitoring of participants likely accounted for the modest weight loss and reduction in SCAT reduction, HbA1c and insulin resistance observed in the placebo group. Consistent with the current study, the EMPA-HEART reported that empagliflozin promoted reverse LV remodelling in patients with diabetes, empagliflozin resulted in a significant reduction in LVMI (−2.6 vs. −0.01 g/m^2^, *P* = 0.01).^28^ It is noteworthy that a recent subgroup analysis of the EMPA-REG OUTCOME trial, reported that the reduction of CVD, MI, and stroke was greater in patients with LVH than in those without LVH, a finding supported by the current study where we found that LVH regression was greater in those with higher baseline LVM.^29^ This suggests that SGLT inhibition may have a greater effect in this higher risk subgroup. Left ventricular hypertrophy regression reduces the incidences of all major CV events; including sudden deaths, heart failure hospitalizations, new onset atrial fibrillation, and strokes independent of BP changes; therefore, our data would suggest that SGLT2i therapy may be warranted for T2D with LVH irrespective of the level of glycaemic control.^30–40^

There are a number of plausible mechanisms that may explain dapagliflozin induced LVM regression some of which have been explored in this study (Take home figure).^41^ Firstly, dapagliflozin could mediate LVH regression through its effect to reduce SBP. Furthermore, there was also a statistically significant correlation between ambulatory SBP reduction and LVM regression that might support this plausible mechanism. Trials have consistently shown that SGLT2i lead to a reduction in SBP in the range of 3–5 mmHg in patients with T2D.^42^ The magnitude of BP reduction was similar to that observed in our study. We also observed that there was a significant drop in nocturnal SBP rather than daytime SBP. The loss of nocturnal decline in BP has been established as an important marker for CV risk, independent of overall BP during a 24-h period.^43^

A second mechanism is reduction in preload secondary to natriuresis and osmotic diuresis which would improve ventricular loading conditions reducing LV wall stress and thus contribute to regression of LVM.^12^ Indeed, mediation analysis from the EMPA-REG OUTCOME trial has suggested that volume contraction is likely a key component of the CV benefit noted in the trial. It has been suggested that ∼50% of the CV benefit seen in the trial could be attributed to empagliflozin induced haemoconcentration.^44^ We did not observe any significant change in NT-proBNP but we did observe a significant increase in haematocrit possibly secondary to decreased plasma volume with resultant haemoconcentration. It is worth noting that the lack of a drop in NT-proBNP may be result of the decrease in body weight.^45^

Obesity is a separate albeit related factor mediating LVH.^15^^,^^46^ A third plausible mechanism for LVH regression seen in this study may be dapagliflozin induced reduction in body weight. Sodium-glucose cotransporter 2 inhibitors have consistently been shown to lead to weight reduction of 2–3 kg.^42^ The weight loss, however, does appear to plateau after 3–6 months.^47^ In this study, dapagliflozin significantly reduced weight on average by 4 kg and the weight loss was most significant in the first 4–6 months of therapy. The weight loss associated with selective SGLT2 inhibition is likely due to the glucose excretion with associated caloric loss.^48^

In our study, dapagliflozin also resulted in a mean reduction in VAT and SCAT of around 700 and 600 cm^3^, respectively, compared with placebo. Visceral fat is well recognized to be associated with an increased risk of T2D mellitus, CV complications and overall mortality and associated insulin resistance, inflammation, and oxidative stress.^49–52^ Whilst we did not observe a significant change in oxidative stress with no change in myeloperoxidase, we did see a significant reduction in hsCRP which has been seen before in studies with dapagliflozin.^53^^,^^54^ Chronic low-grade inflammation is recognized a key feature in T2D and its complications including diabetic cardiomyopathy. The observed strong correlation between VAT reduction and LVM regression suggests that a reduction in VAT-mediated inflammation may lead to improved CV remodelling.

Finally, SGLT2i-induced glycosuria has been shown to improve β cell function and insulin sensitivity and this improvement in insulin sensitivity could have mediated the LVM regression.^55^^,^^56^ Insulin resistance is thought to contribute to changes in cardiac tissue seen in LVH.^57^ In our study, dapagliflozin treatment resulted in a significant reduction in fasting glucose, fasting insulin, and glycated haemoglobin. Due to time and financial constraints, we did not perform a hyperinsulinaemic euglycaemic clamp, the ‘gold standard’ for the measurement of insulin sensitivity but we did see that dapagliflozin resulted in a significant reduction in HOMA-IR an index for insulin resistance.

### Limitation of the study

This was a single-centre study with relatively small number of people. However, this trial is the first prospective, adequately powered RCT conducted to date, investigating the efficacy of dapagliflozin to regress LVH. Secondly, it is noteworthy that the cardiac MRI analysis was performed by only a single operator that did not allow us to assess inter-observer variability and there is the possible effect of learning on the reported intra-observer variability. Thirdly, the study was statistically powered only for a single outcome and not statistically powered to detect changes in other secondary endpoints. Therefore, inferential between group comparisons for these secondary endpoints is likely to be exploratory rather than definitive. Although there were no statistically significant differences between the two groups, because of the relatively small sample size, we cannot exclude the possibility that some subtle baseline and demographic differences between two groups might have collectively contributed to our results.

## Conclusion and future directions

In conclusion, this study has shown, for the first time in a randomized controlled trial that dapagliflozin treatment significantly reduces LVM compared with placebo in people with T2D, LVH, and controlled BP. This is consistent with the results seen with empagliflozin in EMPA-HEART and these independent reports provide excellent validation for both studies.

Dapagliflozin improved SBP, increased haematocrit and in addition we have shown that dapagliflozin reduced measures of obesity such as body weight, SCAT, and VAT and reduced insulin resistance and markers of inflammation.

The regression of LVM suggests dapagliflozin can initiate reverse remodelling and changes in left ventricular structure that may partly contribute to the reported cardio-protective effects of dapagliflozin.

## Supplementary Material

ehaa419_Supplementary_Material
