# Supplementary material for: A randomized controlled trial of dapagliflozin on left ventricular hypertrophy in people with type two diabetes: the DAPA-LVH trial
Source: Eur Heart J. 2020 Jun 24;41(36):3421–32. doi: 10.1093/eurheartj/ehaa419 (PMC8202417; doi:10.1093/eurheartj/ehaa419)
Supplement: ehaa419_Supplementary_Material [file ehaa419_supplementary_material.docx]

**Supplementary Material**

**Table of Contents**

**Section A. MRI protocols**

**Section B Supplementary Tables**

1. Table S1: An overview of all visits scheduled within the trial
2. Table S2: Changes after 12 months dapagliflozin treatment in LVM and LVM indexed to BSA, Height, Height^1.7^, Height^2.7^ following sensitivity analysis
3. Table S3: Changes in Measured Parameters on Cardiac MRI Analysed as Subgroups Above and Below Median LVMI to BSA after 12 months dapagliflozin treatment
4. Table S4: Changes after 12 months dapagliflozin treatment in blood pressure after adjustment for baseline measurements
5. Table S5: Common SGLT2 inhibitor side effects in the two treatment arms

**Section C Supplementary Figures**

1. Figure S1: DAPA LVH trial hypothesis.
2. Figure S2: Study Flow Chart
3. Figure S3 Trial Consort Diagram
4. Figure S4: Scatter plot showing the effect of Dapagliflozin on ambulatory 24 hour and nocturnal systolic blood pressure
5. Figure S5: Column mean graph with standard deviation error bars showing the effect of Dapagliflozin on VAT and SCAT volumes.
6. Figure S6: Graph showing the effect of dapagliflozin over time on weight.

**Section A. MRI protocols**

**Cardiac MRI Protocol**

Short axis images from the atrio-ventricular ring to the LV apex were acquired using a 2D retrospectively ECG-gated breath hold segmented SSFP cine sequence with retrospective gating. The imaging parameters were repetition time (TR) = 3.34ms and echo time (TE) = 1.46 ms, and 52^o^ flip angle, and the image stack was acquired over a typical (patient size dependent) field of view of 340-380 mm using 6mm slices. The in-plane pixel matrix was 216x256 and parallel imaging (i-PAT factor 2) was applied, along with bandwidth 977 Hz/pixel. Images were typically acquired at a rate of two slices per breath hold (scan times approximately 10-15 seconds) although this was reduced to a single slice per breath hold if a shorter scan time was necessary. The images were exported and the analysis was performed off line sequence using CVI 42 (Circle Cardiovascular Imaging software, Calgary, Canada) by a single observer who was blinded to randomised therapy. This was done for the quantitative measurement of LVM, EF, end-diastolic volume (EDV), end-systolic volume (ESV), cardiac output (CO) and stroke volume (SV) which were derived by region of interest contours placed around endocardial and epicardial LV borders at end systole and end diastole.

Papillary muscle mass was included in the LVM measurement if the muscle mass was continuous with the myocardial wall. Care was taken to ensure that the slice ranges were matched for the baseline and final datasets even if it meant the removal of basal or apical slices to allow accurate slice matching. This will have meant an underestimation of the absolute LVM in some scans but ensured accurate comparison between the baseline and final scans. LVM was indexed to body surface area, which were calculated using the Mosteller formula and indexed to height, height^1.7^ and height^2.7^.

Short axis images using the same methods and pulse sequences as for the LV images were also obtained from the atrioventricular ring to the base of the atria. Left atrial volumes were derived by region of interest contours placed around the left atrial endocardial borders at end systole again using CVI 42 (Circle Cardiovascular Imaging software, Calgary, Canada). The left atrial appendage and pulmonary veins were excluded from the left atrial volume.

**Reproducibility and Variability of MRI**

We elected to use a single observer to undertake all of the segmentation work in order to optimise the reproducibility since it is widely known that inter-observer variation tends to introduce the greatest variability in these measurements.

Each scan was analysed at least twice to ensure consistency, a third measurement was conducted if the LVM varied by >5%. And the final measurement was taken as the average of the two closest measurements. By recording the mean LVM in each case, this process lead to improved confidence that the actual LVM values (and changes) were correct and representative of genuine change - i.e. not just measurement error. From these paired baseline measurements of LVM we calculated the intra-class correlation coefficient (ICC) to be 0.99 and we obtained an intra-observer test-retest root-mean-square coefficient of variation (RMS CoV) of 1.91%.

**Abdominal MRI Protocol**

An abdominal MRI was performed to assess subcutaneous and visceral abdominal fat mass. Scout images were acquired in order to localise the full structure of the abdomen in the sagittal, coronal and transverse planes, and a 3D dual-echo Dixon Volume Interpolated Breath hold Examination (Dixon VIBE) sequence was subsequently applied. The sequence was applied twice in the axial orientation covering the entire abdomen via two overlapping 3D blocks. The pulse sequence imaging parameters were TR 3.67ms, TE 1.23 and 2.46ms, flip-angle (FA) 9^o^ and bandwidth 1040 Hz/pixel. A total of 166-192 slices (matrix size 169x320) were acquired over a field of view of 390x480 mm, and each slice was 3mm thick. Each acquisition was performed in a single breath hold (typically 10 seconds) with the patient holding their breath at end expiration.

Following the acquisition, the ‘fat-only’ images were visually inspected and any overlapping slices between the two 3D datasets were removed. The remaining images were then combined to form a single dataset using ImageJ (U. S. National Institutes of Health, Bethesda, Maryland, USA) and the resulting file was exported for analysis using Analyze (Version 12.0, Mayo Clinic, Rochester, MN). The analysis was performed by another blinded observer. This was done in three steps, namely (i) application of baseline regions of interest (ROI) to define sub-cutaneous adipose tissue structures (SCAT) and visceral adipose tissue structures (VAT) using a manually chosen signal-intensity threshold; (ii) selection of anterior and posterior segmentation boundaries - ranging from the top of the diaphragm to the tip of the femoral heads; and (iii) manual removal of ‘non-abdomen’ structures (such as the arms) together with other non-visceral structures such as bone marrow, renal cortex and similar soft-tissues. Care was taken to ensure that the slice ranges were as consistent as possible for the baseline and final datasets. Our chosen rule was to ensure that on a per-patient basis the slice ranges at each of the time-points were consistent to within 3 slices (9mm).

For the abdominal adiposity, repeatability data were not derived specifically for this study cohort but the scans underwent rigorous quality analysis prior to inclusion in the final data set as detailed below.

**Abdominal MRI Quality Analysis**

- The images from two participants were considered to be of sub-standard quality, based on problems associated with motion artefacts and uneven water suppression. One of these participants was omitted from the study entirely (reducing the completing participants to n=60) and in the other case it was only possible to report the VAT volume (i.e. SCAT volume was omitted).
- In n=7 of the largest participants, it was not possible to include the entire SCAT volume within the 480mm FOV. In n=2 of these cases the omitted SCAT regions were deemed large enough to render the volumes as potentially unreliable. In the remaining n=5 cases, the omitted SCAT regions were extremely small (estimated to be <5% of the total SCAT volume) and visually consistent across the two time-points. Consequently, these participants were retained within the study.
- Finally, there were n=7 cases where the radiographic overlap between the two slice blocks on the baseline data was not quite sufficient. However, it was a very simple process to adjust the 12 month data analysis in order to exactly match the included abdominal slices across both time-points.

**Section B Supplementary Tables**

| **Visit** | **1 Screening** | ***2 Baseline** | ***3 follow-up** | **4 Follow-up^** | ***5 Follow-up** | ***6 Last visit** | **Annotations** |
| --- | --- | --- | --- | --- | --- | --- | --- |
| **Timeline - weeks** | **0 to -4** | **0**  **Within 4 weeks of screening visit** | **4**†  **(+/- 1 week)** | **17**  **(+/- 4 weeks)** | **34**  **(+/- 4 weeks)** | **(44-52)**  **(+/- 4 weeks)** | * Participants were fasted for these visits.  † At least 3 weeks after commencing study medication    ‡U&E, FBC, LFT, cholesterol, Lipid profile  § HbA1c, FIRI, glucose, NTproBNP, Leptin, hsCRP, Myeloperoxidase, N terminal Procollagen III peptide. A sample to be held for future genetics was also taken at the randomisation visit.  #The MRI scan could be performed ±3 weeks from the baseline visit and may therefore of required a separate visit although this was never required. |
| Informed Consent | X |  |  |  |  |  |  |
| Medical History | X |  |  |  |  |  |  |
| Demographics | X |  |  |  |  |  |  |
| Concomitant Medications | X | X | X | X | X | X |  |
| Physical Examination | X |  |  |  |  |  |  |
| Height & weight | X |  |  |  |  |  |  |
| BP & P | X | X | X | X | X | X |  |
| Temperature | X |  |  |  |  |  |  |
| ECG | X |  |  |  |  |  |  |
| Echo | X |  |  |  |  | X |  |
| Safety Bloods‡ | X | X | X | X | X | X |  |
| Inclusion/Exclusion | X |  |  |  |  |  |  |
| Pregnancy Testing if applicable^Ω^ | X | X | X | X | X | X |  |
| Research Blood Sample § |  | X |  |  |  | X |  |
| Genetic blood sample |  | X |  |  |  |  |  |
| 24 hour BP |  | X |  |  |  | X |  |
| Cardiac & abdominal MRI# |  | X |  |  |  | X |  |
| Waist & hip measurement |  | X | X | X | X | X |  |
| Adjustment of diabetes and BP medication |  | X | X | X | X |  |  |
| Record Adverse Events |  | X | X | X | X | X |  |
| Randomisation |  | X |  |  |  |  |  |
| Dispense Trial Drugs |  | Xπ | X | X | X |  |  |
| Return trial drugs |  |  | X | X | X | X |  |

Table S1 An overview of all visits scheduled within the trial

Table S2 Changes after 12 months dapagliflozin treatment in LVM and LVM indexed to BSA, Height, Height^1.7^, Height^2.7^ following sensitivity analysis

| Variable | Intention to Treat Analysis | | | | Per Protocol Analysis | | | |
| --- | --- | --- | --- | --- | --- | --- | --- | --- |
|  | Dapagliflozin  (n=32) | Placebo  (n=34) | †Difference  (95% CI) | P Value | Dapagliflozin  (n=29) | Placebo  (n=33) | †Difference  (95% CI) | P Value |
| **Primary Outcome** | | | | | | | | |
| Absolute LVM (g) | -4.00 ± 0.87 | -1.09 ± 0.84 | -2.92 (-5.45 to -0.38) | **0.025** | -4.43 ± 0.93 | -1.11 ± 0.86 | -3.33 (-5.98 to -0.67) | **0.011** |
| **Secondary Outcome** | | | | | | | | |
| LVMi BSA (g/m^2^) | -0.62 ± 0.38 | -0.35 ± 0.37 | -0.27 (-1.38 to 0.85) | 0.634 | -0.67 ± 0.42 | -0.37 ± 0.39 | -0.31 (-1.50 to 0.89) | 0.610 |
| LVMI Height (g/m) | -2.37 ± 0.51 | -0.67 ± 0.50 | -1.70 (-3.20 to -0.21) | **0.026** | -2.62 ± 0.55 | -0.68 ± 0.51 | -1.94 (-3.50 to -0.37) | **0.016** |
| LVMI Height^1.7^ (g/m^1.7^) | -1.65 ± 0.36 | -0.48 ± 0.34 | -1.17 (-2.21 to -0.13) | **0.027** | -1.82 ± 0.38 | -0.49 ± 0.35 | -1.33 (-2.41 to -0.24) | **0.018** |
| LVMI Height^2.7^ (g/m^2.7^) | -0.98 ± 0.21 | -0.30 ± 0.21 | -0.68 (-1.30 to -0.06) | **0.031** | -1.08 ± 0.23 | -0.31 ± 0.21 | -0.77 (-1.42 to -0.12) | **0.021** |

P-values in bold indicate P<0.05; † Estimated marginal mean difference between groups**.** All values expressed in estimated marginal mean ± standard error unless stated. Abbreviations: BSA, Body Surface Area; LVM, Left Ventricular Mass: LVMI, Left Ventricular Mass

| Variable | LVMI to BSA Above Median | | | | LVMI to BSA Below Median | | | | P_interaction_ |
| --- | --- | --- | --- | --- | --- | --- | --- | --- | --- |
|  | Dapagliflozin  (n=18) | Placebo  (n=15) | †Difference  (95% CI) | P Value | Dapagliflozin  (n=14) | Placebo  (n=19) | †Difference  (95% CI) | P Value |  |
| Absolute LVM (g) | -5.11 ± 4.87 | -1.23 ± 4.80 | -3.88 (-7.15 to -0.61) | **0.021** | -2.46 ± 4.57 | -1.05± 4.47 | -1.41 (-4.71 to 1.88) | 0.395 | 0.228 |
| LVMi BSA  (g/m2) | -1.02 ± 1.94 | -0.39 ± 1.78 | -0.64  (-2.07 to 0.79) | 0.376 | -0.01 ± 2.64 | -0.38± 2.64 | 0.36  (-1.08 to 1.80) | 0.616 | 0.973 |
| LVMI Height (g/m) | -2.97 ± 2.79 | -0.73 ± 2.70 | -2.24  (-4.18 to -0.31) | **0.024** | -1.50 ± 2.86 | -0.69± 2.74 | -0.81  (-2.76 to 1.14) | 0.408 | 0.303 |
| LVMI Height1.7 (g/m1.7) | -2.03 ± 1.9 | -0.50 ± 1.82 | -1.53  (-2.88 to -0.18) | **0.027** | -1.06 ± 2.07 | -0.51± 1.95 | -0.55  (-1.91 to 0.81) | 0.422 | 0.312 |
| LVMI Height2.7 (g/m2.7) | -1.19 ± 1.10 | -0.30 ± 1.04 | -0.89  (-1.70 to -0.07) | **0.034** | -0.65± 1.31 | -0.34± 1.21 | -0.31  (-1.13 to 0.51) | 0.446 | 0.327 |

Table S3 Changes in Measured Parameters on Cardiac MRI Analysed as Subgroups Above and Below Median LVMI to BSA after 12 months dapagliflozin treatment

P-values in bold indicate P<0.05; †Absolute mean Difference between groups**.** All other values expressed in mean ± SD unless stated. * Median ± IQR

Abbreviations: LVM, Left Ventricular Mass; LVMI, Left Ventricular Mass indexed.

Table S4 Changes after 12 months dapagliflozin treatment in blood pressure after adjustment for baseline measurements

|  | Intention to Treat | | | | Per Protocol | | | |
| --- | --- | --- | --- | --- | --- | --- | --- | --- |
| Variable | Dapagliflozin  (n=32) | Placebo  (n=34) | †Difference  (95% CI) | P Value | Dapagliflozin  (n=29) | Placebo  (n=33) | †Difference  (95% CI) | P Value |
| § 24 hour SBP | -2.58 ± 0.98 | 0.66 ± 0.97 (n=33) | -3.24(-6.01 to -0.47) | **0.023** | -2.90 ± 1.45 | 0.72 ± 0.99 (n=32) | -3.62(-6.52 to -0.72) | **0.015** |
| §24 hour DBP | -0.79 ± 0.77 | -0.09 ± 0.76 (n=33) | -0.70(-2.88 to 1.48) | 0.523 | -0.89 ± 0.84 | -0.07 ± 0.80 (n=32) | -0.89(-2.56 to 0.78) | 0.481 |
| §Daytime SBP | -2.22 ± 1.09 | 0.31 ± 1.07 (n=33) | -2.53(-5.59 to 0.53) | 0.104 | -2.54 ± 1.16 | 0.40 ± 1.1 (n=32) | -2.94(-6.15 to 0.26) | 0.071 |
| §Daytime DBP | -0.84 ± 0.96 | 0.06 ± 0.94 (n=33) | -0.89(-3.59 to 1.81) | 0.511 | -0.95 ± 1.43 | 0.08 ± 0.98 (n=32) | -1.04(-3.91 to 1.83) | 0.472 |
| §Nocturnal SBP | -3.12 ± 1.23 | 0.56 ± 1.23 (n=32) | -3.68(-7.17 to -0.19) | **0.039** | -3.51 ± 1.31 | 0.64 ± 1.27 (n=31) | -4.15(-7.83 to -0.47) | **0.028** |
| § Nocturnal DBP | -1.89 ± 0.85 | -0.20 ± 0.85 (n=32) | -1.69(-4.12 to 0.75) | 0.171 | -2.14 ± 0.91 | -0.16 ± 0.88 (n=31) | -1.98(-4.55 to 0.58) | 0.127 |
| Office SBP | -5.08 ± 1.31 | -1.99 ± 1.27 | -3.09(-6.75 to 0.57) | 0.096 | -5.60 ± 1.39 | -2.05 ± 1.30 | -3.55(-7.36 to 0.27) | 0.068 |
| Office DBP | -2.79 ± 1.12 | -2.40 ± 1.09 | -0.39(-3.52 to 2.74) | 0.804 | -3.07 ± 1.20 | -2.48 ± 1.12 | -0.59(-3.88 to 2.69) | 0.720 |

P-values in bold indicate P<0.05; † Estimated marginal mean difference between groups**.** All values expressed in estimated marginal mean ± standard error unless stated.

‡ One participant unable to tolerate any ambulatory blood pressure monitoring, § One further participant unable to tolerate overnight blood pressure monitoring.

Abbreviations: DBP, Diastolic Blood Pressure; SBP, Systolic Blood Pressure.

| Side Effect | All patients | Dapagliflozin | Placebo | P value |
| --- | --- | --- | --- | --- |
| *Urinary Symptoms | 15 | 8 | 7 | 0.434 |
| †Confirmed UTI | 7 | 1 | 6 | **0.011** |
| Thrush | 14 | 12 | 2 | **0.046** |
| Dizzy/Postural Hypotension | 1 | 0 | 1 | 0.378 |
| $ Reported Hypoglycaemia | 29 | 22 | 7 | **0.059** |
| Thirst | 4 | 2 | 2 | 0.631 |
| Constipation | 6 | 4 | 2 | 0.814 |
| Serious Adverse Event | 5 | 2 | 3 | 0.357 |

Table S5: Common SGLT2 inhibitor side effects in the two treatment arms

*Polyuria/Urinary Frequency

†Positive mid specimen stream urine

‡ Reported hypoglycaemia by patient based on symptoms and or confirmed by bm monitoring

**Section C Supplementary Figures**

**Examination/ Investigation**

**Parameter**

**Outcome**

**Cardiac – MRI**

**Preload**

**Afterload**

**Reduction in LVH**

**Ambulatory BP**

- **EDV/ESV**
- **SV/CO**
- **LVEF**
- **LV Mass**
- **LA Volumes**

**Insulin Resistance and direct SGLT 2 effects**

- **24 Hour** **BP**

**Research Bloods**

- **NTproBNP**
- **FIRI, HBA1_c_**
- **MPO**
- **Leptin**
- **PIIIP**
- **hsCRP**

**Abdominal – MRI**

- **Visceral Fat**
- **Subcutaneous Fat**

**Weight Loss**

Figure S1: DAPA LVH trial hypothesis.

Abbreviations: BP: Blood Pressure; EDV: End Diastolic Volume; FIRI: Fasting Insulin Resistance Index; HBA1_C_: Glycosylated Haemoglobin; hsCRP; High Sensitive CRP; LA: Left Atrial; LV: Left Ventricular; LVEF: Left Ventricular Ejection Fraction; LVH: Left Ventricular Hypertrophy; MPO: Myeloperoxidase; MRI: Magnetic Resonance Imaging; NTproBNP ; N Terminal pro B Type Natriuretic Peptide; PIIIP; Procollagen III .Peptide.

Opportunistic recruitment at Diabetic/Cardiovascular clinics in NHS Tayside or via SDRN or SPCRN

Excluded:

- Did not meet inclusion criteria

**Enrolment**

Excluded:

- Declined to participate

Invited to participate:

Informed Consent

Meets inclusion criteria

Randomisation (n=66)

**Treatment Allocation**

**Group A** – Allocated to Dapagliflozin 10mg (n=32)

**Group B** – Allocated to placebo group (n=34)

Reviews

Safety visits at weeks 4, 17 and 34 and final review at 10-12 months

**Follow-Up**

**Primary Outcome**

- Changes in LV mass

**Secondary Outcome**

- Changes in other cardiac MRI markers
- Changes in BP
- Changes in BMI
- Changes in subcutaneous and visceral fat mass
- Changes in biomarkers
- Assessment of tolerability of dapagliflozin

**Primary Outcome**

- Changes in LV mass

**Secondary Outcome**

- Changes in other cardiac MRI markers
- Changes in BP
- Changes in BMI
- Changes in subcutaneous and visceral fat mass
- Changes in biomarkers
- Assessment of tolerability of dapagliflozin

**Analysis**

Figure S2 Study design flowchart.

SDRN Scottish Diabetes Research Network; SPCRN Scottish Primary Care Research Network; MRI Magnetic Resonance Imaging; BP Blood Pressure; Body Mass Index

Reviews

Safety visits at weeks 4, 17 and 34 and final review at 10-12 months

Figure S3 : Trial Consort Diagram

Total Screened n = 320

Not recruited (n = 254)

No LVH (n = 203)

Raised BP (n = 10)

HbA1_c_ out of range (n = 17)

Patient preference (n = 5)

Claustrophobia (n = 5)

Previous SGLT2 (n = 5)

Other (n = 9)

Randomised to Dapagliflozin (n = 32)

Randomised to placebo (n = 34)

Dropped out (n =3)

- Hyponatraemia
- Unable to obtain holiday insurance
- Claustrophobia

Dropped out (n = 1)

- Breast Cancer

Completed trial (n = 29)

Completed trial (n = 33)

Total Invited n = 1541

473 positive responses

1068 not interviewed

153 unsuitable

Figure S4 Scatter plot showing the effect of Dapagliflozin on ambulatory 24 hour and nocturnal systolic blood pressure

****Figure S5 Column mean graph with standard deviation error bars showing the effect of Dapagliflozin on VAT and SCAT volumes.

Figure S6. Graph showing the effect of dapagliflozin over time on weight.

Abbreviations: FV, Final Visit; RV, Randomisation Visit.
